# Adaptive radiation in extremophilic Dorvilleidae (Annelida): diversification of a single colonizer or multiple independent lineages?

**DOI:** 10.1002/ece3.314

**Published:** 2012-07-16

**Authors:** Daniel J Thornhill, Torsten H Struck, Brigitte Ebbe, Raymond W Lee, Guillermo F Mendoza, Lisa A Levin, Kenneth M Halanych

**Affiliations:** 1 Department of Conservation Science and Policy, Defenders of Wildlife1130 17th St. NW, Washington, District of Columbia, 20036, USA; 2 Department of Biological Sciences, Auburn University101 Rouse Life Sciences Building, Auburn, Alabama, 36849, USA; 3 Department of Biology/Chemistry, AG Zoology, University of OsnabrückBarbarastrasse 11, D-49069, Osnabrück, Germany; 4 Zoological Research Museum Alexander KoenigAdenauerallee 160, D-53113, Bonn, Germany; 5 Alfred-Wegener-Institute for Polar and Marine ResearchAm Handelshafen 12, D27570, Bremerhaven, Germany; 6 School of Biological Sciences, Washington State UniversityPO Box 644236, Pullman, Washington, 99164, USA; 7 Center for Marine Biodiversity and Conservation, Integrative Oceanography Division, Scripps Institution of Oceanography9500 Gilman Drive, La Jolla, California, 92093-0218, USA

**Keywords:** Adaptive radiation, cold seep, deep sea, Extremophile, methane seep, polychaete

## Abstract

Metazoan inhabitants of extreme environments typically evolved from forms found in less extreme habitats. Understanding the prevalence with which animals move into and ultimately thrive in extreme environments is critical to elucidating how complex life adapts to extreme conditions. Methane seep sediments along the Oregon and California margins have low oxygen and very high hydrogen sulfide levels, rendering them inhospitable to many life forms. Nonetheless, several closely related lineages of dorvilleid annelids, including members of *Ophryotrocha*, *Parougia*, and *Exallopus*, thrive at these sites in association with bacterial mats and vesicomyid clam beds. These organisms are ideal for examining adaptive radiations in extreme environments. Did dorvilleid annelids invade these extreme environments once and then diversify? Alternatively, did multiple independent lineages adapt to seep conditions? To address these questions, we examined the evolutionary history of methane-seep dorvilleids using *16S* and Cyt *b* genes in an ecological context. Our results indicate that dorvilleids invaded these extreme habitats at least four times, implying preadaptation to life at seeps. Additionally, we recovered considerably more dorvilleid diversity than is currently recognized. A total of 3 major clades (designated “*Ophryotrocha,*” “Mixed Genera” and “*Parougia*”) and 12 terminal lineages or species were encountered. Two of these lineages represented a known species, *Parougia oregonensis*, whereas the remaining 10 lineages were newly discovered species. Certain lineages exhibited affinity to geography, habitat, sediment depth, and/or diet, suggesting that dorvilleids at methane seeps radiated via specialization and resource partitioning.

## Introduction

The adaptability of life is truly remarkable, as evidenced by the ability of organisms to exist in most environments on Earth. Certain habitats, however, challenge the persistence of life with adverse environmental conditions, such as extreme temperature, pressure, desiccation, pH, radiation, salinity, oxygen concentration, and/or toxins (reviewed in Rothschild and Mancinelli 2001). Biological diversity in these extreme habitats is often limited (e.g., Gough et al. 2000; Tsurumi 2003; Tobler et al. 2006), and yet certain organisms have evolved physiological tolerance, protective structures, repair capabilities, and other mechanisms that enable survival and success under extreme conditions (reviewed in Grieshaber and Völkel 1998; McMullin et al. 2000; Rothschild and Mancinelli 2001). In complex multi-cellular organisms, such mechanisms can be sophisticated, and presumably energetically expensive, implying that adaptation to extreme environments should be rare.

Methane seeps are one example of an extreme environment. Biological assemblages in these ecosystems interact with methane- and sulfide-rich fluid percolating upward through sediments. As water migrates through these sediments, a series of methane-oxidizing and sulfate-reducing microbial reactions transpire, resulting in extremely high sulfide pore-water concentrations (Sahling et al. 2002; Valentine 2002; Levin et al. 2003). Additionally, little dissolved oxygen penetrates into methane-seep sediments due to strong upward fluid flow as well as reaction with sulfides or reduced metals (Tryon et al. 2001; Levin et al. 2003). Because of the high toxicity of sulfide (i.e., levels greater than 1 mmol/L are toxic to most metazoans; Grieshaber and Völkel 1998) and unavailability of dissolved oxygen, methane seeps are among the most physiologically challenging environments for aerobic animals. Typically, species diversity is low at methane seeps (Levin 2005; Cordes et al. 2010; Levin et al. 2010), but several taxa may have radiated within seeps, including dorvilleid, ampharetid, hesionid, siboglinid, and polynoid annelids as well as vesicomyid clams (reviewed in Sibuet and Olu 1998; Levin 2005).

**Figure 1:**
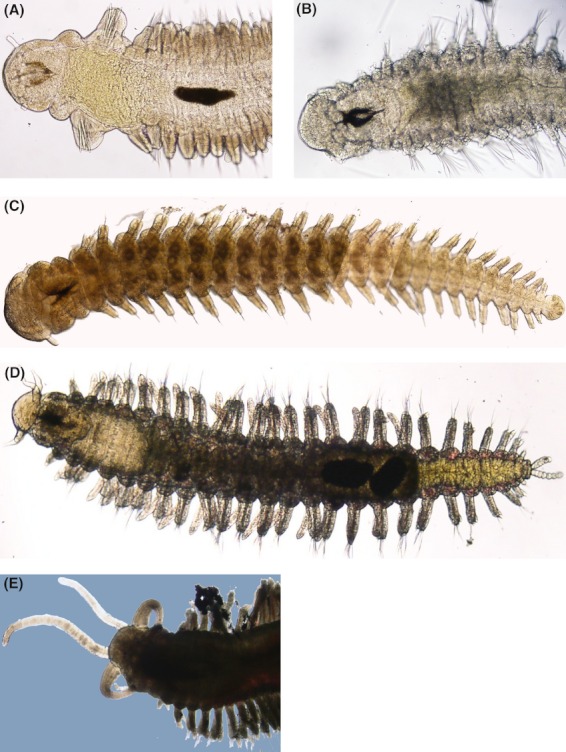
Light micrographs depicting dorvilleid annelids from methane seeps on the NE Pacific margin. Taxa depicted include (A) an undescribed *Exallopus* sp. (designated as *Exallopus* Seep in Figs. 4), (B) an undescribed *Ophryotrocha* sp. that resembled *O. maciolekae* (designated *Ophryotrocha* Seep 1), (C) an undescribed *Ophryotrocha* sp. that resembled *O. platykephale* (designated *Ophryotrocha* Seep 3), (D) an undescribed *Parougia* sp. (designated as *Parougia* Seep Clade OR), and (E) *Parougia oregonensis* (designated as *P. oregonensis* Clade 1). Images are not to scale with one another. Photo credits T.H. Struck.

Previous studies characterizing diversity of methane seep fauna have, understandably, given considerable attention to large symbiotic taxa including tube worms, vesycomid clams, and *Bathymodiolus* spp. mussels as well as archeal and eubacterial communities that are critical to ecosystem function (reviewed in Sibuet and Olu 1998; Levin 2005). At methane seeps (500–880 m deep) off northern California and Oregon, dorvilleid polychaetes are the dominant macrofauna in microbial-mat-covered sediments, and are abundant in vesicomyid clam beds, ampharetid beds, and on authigenic carbonates rocks (Fig. 1; Sahling et al. 2002; Levin et al. 2003, 2010; Thurber et al. 2009, 2012). These animals are most concentrated in sediments with sulfide concentrations of 1–5 mmol/L, where they achieve remarkably high densities (reaching greater than 11,000 individuals per square meter; Levin et al. 2003). Furthermore, the majority of seep-dwelling dovilleids are new to science (Levin et al. 2003, 2010). Three factors make this system unusual: (1) many different species coexist in the same sediments, (2) a single annelid family comprises most of the macrofauna, and (3) high densities of animals thrive at very high-sulfide concentrations. Given these factors, dorvilleids at the Cascadian margin methane seeps provide a suitable system to address questions about evolution at physiologically challenging environments. Hypothetically, the exceptional tolerance to low-oxygen and high-sulfide concentrations of dorvelleid annelids has allowed this group to exploit ecological niches that are unavailable to most organisms. Over evolutionary time, absence of predators and competitors at western North American methane seeps could function as an evolutionary release, facilitating diversification (Levin et al. 2003). Whether this diversification occurred following colonization by a single lineage or multiple-independent colonization events is a key question considered here.

“Dorvilleidae” is an old and diverse polychaete assemblage within Eunicida, comprising at least 33 genera, including *Exallopus*, *Parougia*, *Pinniphitime*, *Pseudophryotrocha*, and the speciose *Ophryotrocha* (Struck et al. 2006, 2007). Dorvilleids occupy a diverse range of habitats and are often opportunistic infauna that are abundant in eutrophic and early-successional environments (Thornhill et al. 2009). These worms are also found in highly reduced and sulfidic extreme environments, including hydrothermal vents, whale-fall sediments, and cold methane seeps in the deep sea (Bernardino et al. 2010). Despite the group's diversity and abundance, only four phylogenetic studies have been conducted within dorvilleids (i.e., Pleijel and Eide 1996; Dahlgren et al. 2001; Heggøy et al. 2007; Wiklund et al. 2009), all of which focus on the numerous *Ophryotrocha* species from nonseep environments. Inferred relationships among shallow-water and whale-fall *Ophryotrocha* species were by and large congruent between molecular phylogenetic studies (Dahlgren et al. 2001; Heggøy et al. 2007; Wiklund et al. 2009). Conversely, Pleijel and Eide's (1996) morphological analysis suggested a markedly different ophryotrochan phylogeny (reviewed in Thornhill et al. 2009). Genetic data generally supported a gonochoristic *labronica* group and a second clade consisting of the hermaphroditic *Ophryotrocha* species (reviewed in Thornhill et al. 2009; see also Wiklund et al. 2009 where additional clades of hermaphroditic *Ophryotrocha* were reported). Furthermore, Heggøy et al. (2007) noted that *Ophryotrocha* was paraphyletic as *Iphitime paguri* fell within *Ophryotrocha*. None of these studies included species from hydrothermal-vent or methane-seep settings, where sulfide levels are higher and taxa are more ubiquitous.

Herein, we investigate the adaptive radiation of animals in extreme environments using methane-seep dorvilleid annelids in the northeast Pacific as a study system. The nature of dorvilleid diversification in these habitats provides insight into colonization of, and adaptation to, extreme environments. Specifically, if dorvilleids radiated only after moving into seep environments, adaptations required for life in extreme environments would be assumed to rarely evolve, because dorvilleids overcame physiological challenges of extreme environments only once during their evolutionary history. The single invasion of seeps by dorvilleids is our null hypothesis. By contrast, if dorvilleids radiated prior to (as well as after) colonizing seep habitats, the ability to adapt to such environments would be inferred to have occurred numerous times and with relative ease over evolutionary time. To determine evolutionary origins of methane seep dorvellids, we examined 16S rRNA (*16S*) and cytochrome b (Cyt *b*) mitochondrial gene sequence data. We also examined dorvilleid diversification and the coexistence of multiple species in relation to substrate depth, habitat type, food source data as inferred by δ^13^C, and geographic location.

## Methods

### Sample collection

Dorvilleid annelids were collected from depths of 590–900 m on the northern California continental slope off shore of the Eel River mouth and on the Oregon margin at Hydrate Ridge (Table 1). Collections took place during three research cruises aboard the *R/V Western Flyer* in July 2005 and *R/V Atlantis* in July 2006 and October 2006. Sediment samples containing methane-seep infauna were taken using 30-cm long, 8.3-cm diameter tube cores or scoop bags using the remotely operated vehicle *Tiburon* (July 2005) or deep-sea submersible vehicle *Alvin* (July and October 2006). Methane-seep habitats sampled included vesicomyid*-*clam aggregations and microbial mats. Habitats, some with active venting of methane bubbles, were identified following Levin et al. (2003, 2010). Additional habitats, including tube fields and carbonate deposits, were also sampled when available. Once returned to the surface, samples were stored at 5–6°C (ambient bottom-water temperature) and processed immediately by sectioning the tube cores vertically at 0–1, 1–2, 2–5, and 5–10 cm depths. Fauna was subsequently sorted following Levin et al. (2003). Photographs depicting a representative sub-set of NE Pacific margin dorvilleid diversity are provided in Figure 1. Morphologically identified dorvilleids were either (1) frozen or preserved in 85% ethanol for molecular analyses (*n* = 131), (2) frozen for analyses of stable isotopic signatures, or (3) preserved in formalin as voucher specimens based on morphological assessments made on board the ship. Sediment position and habitat type were documented for each specimen. Because not all collected dorvilleid worms could be placed in a recognized species, some were assigned temporary epitaphs based on morphological and molecular characterizations. Novel lineages mentioned here will be assigned official names as part of a larger ongoing effort in Dorvilleidae taxonomy.

**Table 1:** Sampling locations by region, site name, geographic coordinates, and depths of collection

Region	Site	Latitude	Longitude	Depth (m)
Eel River, California	North	N 40°48.7′	W 124°36.7′	514
	South	N 40°47.1′	W 124°35.8′	523
Hydrate Ridge, Oregon	North	N 44°40.2′	W 125°5.9′	588–609
	South	N 44°34.2′	W 125°8.9′	770–775
	East	N 44°34.3′	W 124°59.9′	872–880

### DNA extraction, PCR, and sequencing

Genomic DNA was extracted using the DNeasy Tissue Kit (Qiagen Inc., Valencia, CA) or a standard Hexadecyltrimethylammonium bromide (CTAB) protocol (Doyle and Doyle 1987). Due to the small size of most specimens (<1 mm length; Fig. 1), a whole-genomic amplification step (using a GenomiPhi kit from GE Healthcare, Little Chalfont, Buckinghamshire, U.K.) was included when necessary. 339–359-bp fragments of *16S* and 398–403-bp fragments of Cyt *b* were amplified using the primers “16SarL” (5′-CGCCTGTTTATCAAAAACAT-3′) and “16SbrH” (5′-CCGGTCTGAACTCAGATCACGT-3′) for *16S* (Palumbi et al. 1991), and “Cyt b-424F” (5′-GGWTAYGTWYTWCCWTGRGGWCARAT-3′) and “Cyt b-876R” (5′-GCRTAWGCRAAWARRAARTAYCAYTCWGG-3′) for Cyt *b* (von Nickisch-Rosenegk et al. 2001). Polymerase chain reaction (PCR) cycling conditions were as follows: initial denaturation at 94°C for 2 min; 35 cycles of denaturation at 94°C for 30 sec; annealing at 45°C for 30 sec (*16S*) or 1 min (Cyt *b*); extension at 68°C for 1 min; final extension at 68°C for 7 min. PCR products were verified by gel electrophoresis.

Purified PCR products were bi-directionally sequenced using a Beckman CEQ 8000 Genetic Analysis System (Beckman Coulter, Brea, CA). Cyt *b* sequences were translated (*Drosophila*-mitochondrial code) into MacClade Version 4.06 (Maddison and Maddison 2000) to ensure that stop codons were not present. Each unique dorvilleid mitochondrial haplotype sequence was designated by a number for Cyt *b*, a lowercase letter for *16S*, and alphanumeric combined name for the concatenated data (Table S1).

### Phylogenetic analyses

Based on results of Struck et al. (2006), *Marphysa* sp. was selected as an outgroup taxon. Nucleotide sequences were aligned automatically using Clustal X (Thompson et al. 1997) and manually corrected by eye using SeAl Version 2.0a11 (http://tree.bio.ed.ac.uk/software/seal/) and MacClade version 4.06. For the *16S* alignment, nonseep dorvilleid sequences from Dahlgren et al. (2001) were also included (GenBank accession numbers: AF321419–AF321436, AF380115). Nucleotide positions that could not be unambiguously aligned were excluded from these analyses (Cyt *b n* = 0; *16S n* = 125, concatentated data *n* = 125).

For all analyses conducted herein, Cyt *b* and *16S* data were examined both separately and as a concatenated dataset. Topologies were constructed under Bayesian inference (BI) using MrBayes Version 3.12 (Huelsenbeck and Ronquist 2001) implementing the Hasegawa–Kishino–Yano (HKY) + Γ (Cyt *b*) or General-Time-Reversible (GTR)+I+ Γ (*16S* and concatenated data) models of substitution, as suggested by the hierarchical Likelihood Ratio Test and the Akaike Information Criterion by MrModeltest v2 (Nylander 2004). For each analysis, two sets of four chains (three hot, one cold) were run for 2.0 × 10^6^ generations and sampled every 100 generations. Due to convergence of chains within 1.2 × 10^5^ (Cyt *b*), 9.0 × 10^4^ (*16S*), and 1.5 × 10^5^ generations (concatenated), the first 1,200 (Cyt *b*), 900 (*16S*), or 1,500 (concatenated) trees were discarded as burn-in, and a 50% majority-rule consensus tree was calculated from remaining trees. Posterior probabilities (PP) were recorded to assess reliability of recovered nodes.

Maximum Likelihood (ML) analyses were also conducted with PAUP4.0 (Swofford 2002) for all three datasets using the same substitutions models as in the BI and fixed-model parameters as indicated by MrModeltest. Heuristic searches were run with random-taxon addition (10 replicates) and Tree-Bisection-Reconnection (TBR). Robustness of the nodes was determined by 100 bootstrap replicates using RAxML version 7.0.4 at the RAxML black box (http://phylobench.vital-it.ch/raxml-bb/; Stamatakis et al. 2008).

Topology tests using the AU test of CONSEL (Shimodaira and Hasegawa 2001; Shimodaira 2002) were performed under the ML criterion to compare several hypotheses against the best tree. The following hypotheses, if appropriate for the dataset, were tested: (1) monophyly of *Ophryotrocha* Seep 1 and 2 (to assess plasticity of the *O. maciolekae*-like phenotype that these lineages possess) (*16S*); (2) monophyly of *Ophryotrocha* Seep 3–5 (to assess plasticity of the *O. platykephale*-like phenotype that these lineages possess) (Cyt *b*); (3) monophyly of *Ophryotrocha* Seep 1–5 (Cyt *b*, *16S*); (4) monophyly of *Ophryotrocha* (Cyt *b*, *16S*, concatenated); (5) monophyly of *Ophryotrocha* Seep 1–5 plus *Exallopus* and *Pinniphitime* (Cyt *b*), monophyly of *Ophryotrocha* Seep 1–5 plus *Exallopus* (concatenated); or (6) monophyly of *Ophryotrocha* Seep 3–5, *Pseudophryotrocha* and *Exallopus* plus *Parougia* and *D. albomaculata* (*16S*). To obtain the best result for each hypothesis, the analyses were constrained by allowing only trees congruent with the particular hypothesis in heuristic searches in PAUP4.0 using the same settings as above.

### Isotopic diet analyses

Based on the morphological and molecular identifications of dorvilleid specimens, 4–62 individuals of each clade were analyzed for tissue δ^13^C. Specimens were rinsed in MilliQ water, dried, powdered, and homogenized (when necessary), placed in tin boats, and acidified with 10% PtCl_2_ to remove carbonate. Specimens were analyzed on a Costech elemental analyzer with a “zero-blank” autosampler interfaced with a continuous-flow Micromass Isoprime isotope-ratio mass spectrometer at Washington State University or on a Finnigan Conflow 2 continuous-flow system and a Fisons NA 1500 elemental analyzer coupled to a Finnegan Delta S isotope-ratio mass spectrometer at Boston University. Isotope ratios are expressed as δ^13^C in per mil units (‰). Standards for ^13^C were PeeDee Belemnite.

## Results

The molecular data set consisted of 131 total methane-seep dorvilleid samples (*n* = 130 Cyt *b*, *n* = 128 *16S*, *n* = 127 samples sequenced for both genes). These were grouped into 41 unique haplotypes for Cyt *b* and 18 haplotypes for *16S*. The number of representatives per haplotype ranged from 1 to 24 for Cyt *b* and from 1 to 38 for *16S* (Table S1, Figs. 4). When these data were concatenated into a combined dataset, a total of 43 unique haplotypes were encountered. This higher number relative to the individual genes reflects the fact that the unambiguously aligned region of *16S* is more conserved than Cyt *b* (Mueller 2006); many samples of the same *16S* haplotype exhibited Cyt *b* nucleotide differences. Concatenation was not possible for the “*Pinniphitime* Seep” and “*Pseudophryotrocha* Seep” samples, which were successfully sequenced for only one gene each (Table S1). The number of representatives per haplotype ranged from 1 to 23 for the concatenated data.

**Figure 2:**
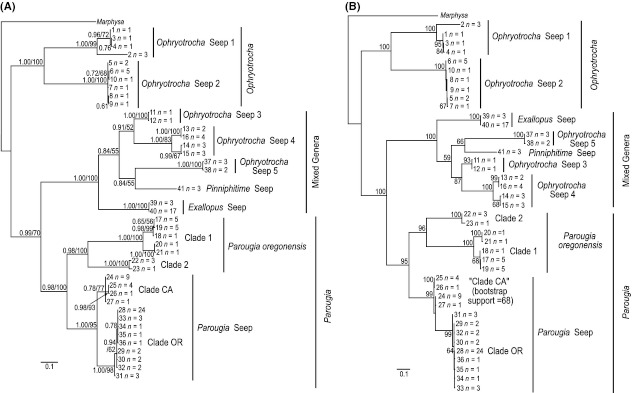
Bayesian inference topology (A) and one of the two best trees of the Maximum Likelihood analysis (B) based on Cyt *b* mtDNA of dorvilleid polychaetes from methane seeps of the Cascadian margin. The second ML tree differed only within *Ophryotrocha* Seep 2 ingroup relationships. For the Bayesian inference, nodal support indicated as posterior probabilities or bootstrap values (numerical values) above 0.50 or 50, respectively, next to the relevant node. Bootstrap values are provided next to the relevant node in the Maximum Likelihood analysis. For each haplotype, the haplotype name (Arabic numerals corresponding to Table S1) and number of replicates (designated as ‘*n*=’) are provided. For described species, the species name is provided to the right of the phylogeny. Undescribed species are each labeled by their putative genus (identified based on morphological characters) and a tentative cladal designation (e.g., *Ophryotrocha* Seep 1). Major groupings on the phylogeny (i.e. *Ophryotrocha*, Mixed Genera, and *Parougia*) are also labeled.

**Figure 3:**
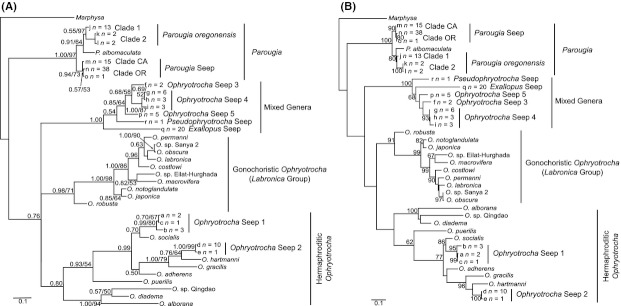
Bayesian inference topology (A) and the best Maximum Likelihood tree (B) based on *16S* mtDNA of dorvilleid polychaetes from methane seeps of the Cascadian (CA and OR) margin as well as *Ophryotrocha* spp. from nonseep environments. For the Bayesian inference, nodal support indicated as posterior probabilities or bootstrap values (numerical values) above 0.50 or 50, respectively, next to the relevant node. Bootstrap values are provided next to the relevant node in the Maximum Likelihood analysis. For each haplotype, the haplotype name (lowercase letters corresponding to Table S1) and number of replicates (designated as ‘*n*=’) are provided. For described methane seep species, the species name is provided to the right of the phylogeny. Undescribed seep species are each labeled by their putative genus (identified based on morphological characters) and a tentative cladal designation (e.g., *Ophryotrocha* Seep 1). Nonseep species are labeled following Dahlgren et al. (2001). Major groupings on the phylogeny are also labeled.

**Figure 4:**
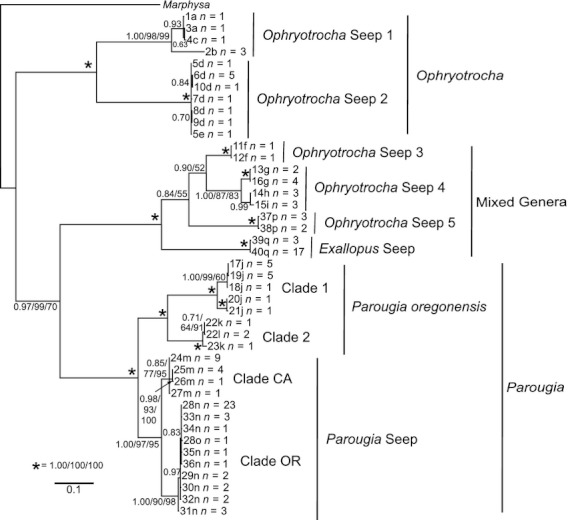
Maximum Likelihood topology based on concatenated Cyt *b* and *16S* mtDNA genes of dorvilleid polychaetes from methane seeps of the Cascadian margin. Nodal support values (above 0.50 or 50) are indicated next to the relevant node as posterior probabilities of the BI topology (at the first position or alone), bootstrap values of the ML tree (at the second position), and ML bootstrap analysis values of the BI topology (at the third position). Alphanumeric names (designated by letters corresponding to Cyt *b* haplotype and numbers corresponding to *16S* haplotype) and number of replicates (designated as ‘*n* = ’) are provided for each haplotype. For described species, the species name is provided to the right of the phylogeny. Undescribed species are each labeled by their putative genus (identified based on morphological characters) and a tentative cladal designation (e.g., *Ophryotrocha* Seep 1). Major groupings on the phylogeny (i.e., *Ophryotrocha*, Mixed Genera, and *Parougia*) are also labeled.

Alignments had a total of 403 (Cyt *b*), 387 (*16S*; with the addition of 19 nonseep dorvilleid taxa), and 790 (concatenated) positions with 403 (Cyt *b*; 100%), 262 (*16S*; 67.7%), and 665 (concatenated; 84.2%) unambiguously aligned positions used in analyses, respectively. For Cyt *b*, *16S*, and concatenated datasets, respectively, there were 240, 199, and 410 variable positions, with 225, 191, and 409 of these being phylogenetically informative.

Topologies of methane seep dorvilleids, as estimated by BI and ML, are shown in Figs. 4. Overall, the two mitochondrial genes yielded similar estimations of evolutionary relationships among taxa. However, the Cyt *b* topology exhibited relatively longer branch lengths (reflecting more substitutions per site in this gene between taxa) than was observed for *16S*. Specific patterns within the three topologies are highlighted below.

### Cytochrome b mtDNA

Topology (Fig. 2) based on Cyt *b* resolved at least 11 well-supported terminal clades of dorvilleids, with posterior probabilities generally above 0.95 and bootstrap values above 95. These 11 terminal clades formed three major groups (labeled as “*Ophryotrocha*,” “Mixed Genera,” and “*Parougia*” in Fig. 2). The “*Ophryotrocha*” group (PP = 1.00; BP = 100) contained two terminal clades – *Ophryotrocha* Seep 1 and Seep 2 (both PP = 1.00; BP = 100). The second group of methane seep dorvilleids, “Mixed Genera” (PP = 1.00; BP = 100), included three *Ophryotrocha* lineages (designated *Ophryotrocha* Seep 3, 4, and 5 in Fig. 2), which were generally well supported (PP = 1.00; BP ≥93). This group also included lineages representing *Exallopus* and *Pinniphitime* species. This group was most closely associated with the third group “*Parougia*” (PP = 0.99; BP = 100), which included *Parougia oregonensis* (PP ≥0.98; BP = 96) and an undescribed *Parougia* (PP = 1.00; BP = 99; Fig. 1), both of which split into two lineages. Clade 1 and 2 within *P. oregonensis* as well as clade OR of *Parougia* Seep received strong support (PP = 1.00; BP ≥99), while nodal support for the clade CA was weak and monophyly was only recovered in BI (PP = 0.78; BP = 68; Fig. 1). Notably, the CA and OR clades were partitioned geographically from one another at the Eel River and Hydrate Ridge sites, respectively (Table 2, Table S1; although we note the detection of a single specimen of *Parougia* Clade CA at Hydrate Ridge).

**Table 2:** Geographic distribution, habitat type, sediment depth, and dietary data that ecologically differentiate the dorvilleid taxa examined in this study

Dorvilleid Taxon/Clade^1^	Geographic distribution^2^	Habitat type	Mean sediment depth (cm)	Sediment depth Range (cm)
*Exallopus* Seep	ER-NR,SRHR-N,S	Bacterial mat,Clam bed,Tube field	4.24	0–10
*Ophryotrocha* Seep 1 and 2^3^	ER-NR,SR	Bacterial mat^4^, Clam bed, Carbonate field	1.62	0–5
*Ophryotrocha* Seep 3	HR-E	Bacterial mat	1.75	0–5
*Ophryotrocha* Seep 4 and 5	ER-NR,SR	Bacterial mat^4^, Clam bed	0.96	0–7
*Parougia oregonensis* Clades 1 and 2^3^	ER-NR,SRHR-N,S,E	Bacterial mat,Clam bed^4^,Tube field	1.64	0–7
*Parougia* Seep CA	ER-N,SHR-S^5^	Bacterial mat^4^,Clam bed	1.47	0–3
*Parougia* Seep OR	HR-N,S,E	Bacterial mat,Clam bed^4^,Tube field	1.61	0–10
*Pinniphitime* Seep	ER-NHR-N,S	Bacterial mat,Clam bed	NA^6^	NA^6^
*Pseudophryotrocha* Seep	ER-N	Bacterial mat^4^, Clam bed	NA^6^	NA^6^

1 Corresponding to the phylogenies presented in Figs. 4

2 Collection site abbreviations are as follows: ER, Eel River; California; NR, North Ridge; SR, South Ridge; HR, Hydrate Ridge, Oregon; N, North; S, South; E, East.

3 Sub-clades within this group were unable to be differentiated morphologically or geographically, and thus data have been pooled here.

4 Indicates that this dorvilleid clade was most abundant in this habitat and rare in the other habitats.

5 A single specimen from this clade was encountered at HR-S; all other individuals were encountered at ER.

6 Data not available.

### 16S ribosomal mtDNA

Similar to Cyt *b* results, approximately 11 lineages of methane-seep dorvilleids were detected in the *16S* dataset (Fig. 2). These terminal clades were either well supported or represented by only a single haplotype in the dataset. Notably, several clades that were differentiated by Cyt *b* were only distinguished by short branch lengths in the *16S* topology (i.e., *P. oregonensis* Clades 1 and 2; *Parougia* Clades CA and OR; Fig. 3). In most cases, similar relationships between clades were inferred based on *16S* versus Cyt *b*. Within the “Mixed Genera” group, associations were still observed between *Ophryotrocha* clades (Seep 3, 4, and 5) and specimens from other genera, including *Exallopus* and *Pseudophryotrocha* (PP = 1.00; BP = 100). The BI and ML topologies were incongruent only in the placement of the latter two genera within “Mixed Genera” (Fig. 3). In contrast to the affiliation between the “*Parougia*” and “Mixed Genera” groups in the Cyt *b* topology, the “*Ophryotrocha*” and “Mixed Genera” groups were sister to one another, but poorly supported (PP = 0.76; BP >50).

Addition of *16S* data from nonseep *Ophryotrocha* species (Dahlgren et al. 2001) provided additional insight into the evolution of methane-seep dorvilleids. Three major ophryotrochan groups were recovered including a gonochoristic *labronica* group (PP = 0.98; BP = 91), a second group of hermaphroditic species plus two *Ophryotrocha* clades from seeps (PP = 0.80; BP <50), and “Mixed Genera” as the third group. Moreover, *Ophryotrocha* Seep 1 and Seep 2 were not closely related. *Ophryotrocha* Seep 2 was closely affiliated to *O. hartmanni*, *O. gracilis*, and *O. adherens,* whereas *Ophryotrocha* Seep 1 was affiliated to *O. socialis*. Similarly, nonseep *Parougia albomaculata* was nested within the clade of methane-seep *Parougia* species.

### Concatenated mtDNA genes

With the exception of the missing samples representing the *Pinniphitime* (Cyt *b*), *Pseudophryotrocha* (*16S*), nonseep *Ophryotrocha* (*16S*), and *P. albomaculata* (*16S*) lineages, the topology based on concatenated data was highly consistent with topologies produced by individual genes (Figs. 4). Furthermore, results of BI and ML analyses were congruent. Therefore, we present only the ML tree (Fig. 4). Within three major groups, “*Ophryotrocha*,” “Mixed Genera,” and “*Parougia*,” approximately 10 terminal clades of methane seep dorvilleids were detected. Support values for these groups were high for 7 of 10 lineages, with posterior probabilities and bootstrap values above 0.95 and 95, respectively. The remaining three clades – including *Ophroytrocha* Seep 4, *Parougia* Seep Clade CA and Clade OR – had moderate-to-high support, with posterior probabilities ≥0.85 and bootstrap values ≥77.

### Topology testing

We tested alternative hypotheses that were not recovered by the best tree using the AU test. Monophyly of a group comprising all *Ophryotrocha* taxa and no other taxa were significantly rejected by all three datasets (*P* ≤ 0.010) (Table 3). For the *16S* dataset, monophyly of *Ophryotrocha* Seep taxa was also significantly rejected (*P* < 0.001). Additionally, monophyly of *Ophryotrocha* Seep 1 and 2 resembling *O. maciolekae* is significantly different from the best tree in the *16S* dataset (*P* < 0.001). The other two datasets are not appropriate to test this hypothesis due to the lack of hermaphroditic ophryotrochans. In contrast, monophyly of *Ophryotrocha* Seep 3–5 resembling *O. platykephale* to the exclusion of *Pinniphitime* Seep in the Cyt *b* dataset cannot be rejected (*P* = 0.229). This monophyly was given in the *16S* and concatenated datasets, but these datasets lacked *Pinniphitime* sp. Finally, the three datasets recover different placements of the “Mixed Genera” group. *16S* analyses grouped this clade with “*Ophryotrocha*,” whereas other analyses grouped it with “*Parougia*.” However, no dataset was able to reject the alternative scenarios for the placement of this “mixed” group (Table 3).

**Table 3:** Results of topology testing using the AU test of different alternative hypotheses not recovered by the best tree for the three datasets. Significant values (*P* < 0.05) are in bold

Hypothesis	Cyt *b*	16S	Concatenated
Monophyly of “Mixed Genera” and “*Parougia*”	n.a.^1^	0.501	n.a.^1^
Monophyly of “Mixed Genera” and “*Ophryotrocha*”	0.097	n.a.^1^	0.227
Monophyly of *Ophryotrocha*	**0.003**	**0.010**	**0.001**
Monophyly of *Ophryotrocha* Seep 1 and 2	n.a.^2^	**<0.001**	n.a.^2^
Monophyly of *Ophryotrocha* Seep 3–5	0.229	n.a.^1^	n.a.^1^
Monophyly of *Ophryotrocha* Seep 1–5	n.a.^3^	**<0.001**	n.a.^3^

1 Not applicable = Recovered by best tree.

2 Not applicable = Recovered by best tree and dataset is not appropriate due to lack of hermaphroditic “*Ophryotrocha*.”

3 Not applicable = the same as the hypothesis “Monophyly of *Ophryotrocha*.

### Ecology of cold-seep dorvilleids

Several dorvilleids reported herein exhibited differences in their geographic distributions. For instance, two *Parougia* clades (Seep CA and Seep OR) were commonly partitioned geographically. *Parougia* Seep CA was generally found at the Eel River, California sites (note the detection of one specimen of *Parougia* Seep CA at Hydrate Ridge), whereas *Parougia* Seep OR was restricted to Hydrate Ridge (Table 2). Similarly, several clades resembling *Ophryotrocha* displayed limited distributions (Table 2). *Ophryotrocha* Seep 1, 2, 4, and 5 were found solely at Eel River. Conversely, *Ophryotrocha* Seep 3 occurred only at Hydrate Ridge East. However, the sample size is low for certain clades and more exhaustive sampling could uncover broader distributions.

Within each methane seep, several different habitat types were observed, including clam beds, bacterial mats, ampharetid-tube fields, and carbonate deposits. Although virtually no oxygen penetrated into bacterial–mat sediments, clam-bed sediments were penetrated by oxygen in the first few millimeters (Levin et al. 2003). Sulfide concentration also varied by location and habitat type. The sulfide concentrations were highest in bacterial mats at Hydrate Ridge (Sahling et al. 2002; Levin et al. 2003). By comparison, clam beds at Hydrate Ridge and bacterial mats at Eel River exhibited approximately one order of magnitude lower sulfide concentrations (Sahling et al. 2002; Levin et al. 2003; Ziebis and Haese 2005). The lowest sulfide levels occurred in Eel River clam beds (Sahling et al. 2002; Levin et al. 2003; Ziebis and Haese 2005).

Despite occurrence of different habitats, there was little absolute partitioning of dorvilleid clades by habitat (Table 2). Note that ampharetid-tube fields were poorly sampled and less common than other habitats at these seeps. Some seep dorvilleids are commonly found in several different habitats and therefore appear to be methane-seep habitat generalists (e.g., *Exallopus* Seep, *Pseudophryotrocha* Seep, *Pinniphitime* Seep). Nevertheless, certain clades/species were more abundant in one habitat. Specifically, *P. oregonensis* and *Parougia* Seep OR were most abundant in clam beds relative to other habitat types. By contrast, *Parougia* Seep CA and all *Ophryotrocha* clades were dominant in the bacterial-mat habitats of Eel River or Hydrate Ridge East, respectively. Whether these differences represent actual habitat affiliations, as opposed to differences between sites, differential sulfide tolerance between taxa, or geographic partitioning of these species, remains to be determined.

At finer spatial scales, most dorvilleid clades were concentrated in uppermost sediment layers at methane seeps (approximately 0.96–1.75 cm; Table 2). A notable exception was *Exallopus* Seep, which exhibited a broader sediment-depth distribution. *Exallopus* Seep was found at ≤10-cm depth, with individuals being most abundant at approximately 4–5 cm below the sediment surface (Table 2). These worms also had high sulfide tolerance (worms occurred at sulfide concentrations >10 mmol/L; data not shown).

**Figure 5:**
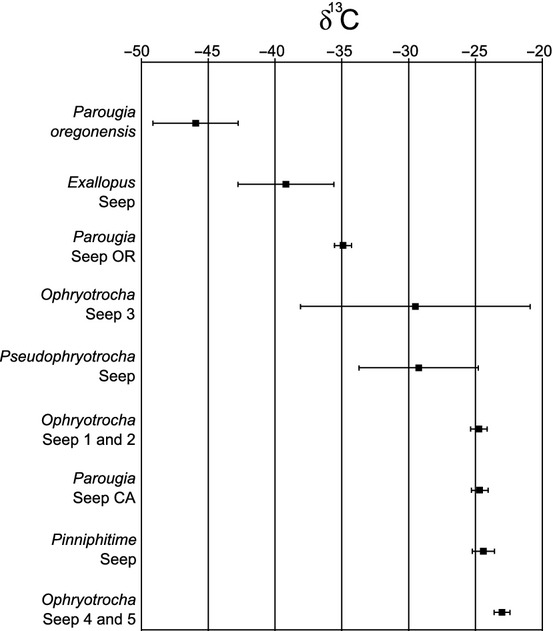
Mean δ^13^C stable isotope values in per mil units for the dorvilleid taxa examined in this study. Error bars represent one standard error for each taxon.

Finally, partitioning among lineages is possibly driven by food sources. Thus, diets of methane-seep dorvilleids were inferred via measurement of carbon stable isotope ratios. For δ^13^C, values near −20‰ reflect photosynthetic food sources, whereas much lighter values reflect chemosynthetic food sources. Values between −25 and −40‰ probably indicate carbon fixed by sulfur oxidation and values of approximately −45‰ and below reflect methane-derived carbon (Fisher 1990; Summons et al. 1998; Van Dover 2000; Levin and Michener 2002). Based on these considerations, *Parougia* Seep CA had δ^13^C values indicative of photosynthetically derived carbon (Fig. 5). Its congeners had lower δ^13^C values; *Parougia* Seep OR appeared to derive its carbon from sulfur oxidation and *P. oregonensis* had values consistent with methane as a carbon source (Fig. 5). The *Exallopus* clade also had low δ^13^C values intermediate between methane-derived and sulfur-oxidation-derived carbon (but note the deeper sediment distribution (Table 2) and higher sulfide tolerance [>10 mmol/L vs. <1 mmol/L sulfide] of *Exallopus* Seep vs. *P. oregonensis*). Remaining clades of *Ophryotrocha*, *Pinniphitime*, and *Pseudophryotrocha* all had heavier δ^13^C values reflecting photosynthetic and/or sulfur oxidation as potential carbon sources.

## Discussion

### Diversity of dorvilleids at methane seeps

Cold methane seeps off of the U.S. Pacific Northwest host highly diverse dorvilleid assemblages, consisting of at least 12 mtDNA species (terminal clades) in 5 different genera. Although two of these clades represented a known nominal species (i.e., *P. oregonensis*), most of the dorvilleid lineages reported here are new to science. By comparison, the mtDNA sequence divergences observed in this study are equivalent to or greater than the genetic distances reported for different shallow-water dorvilleid species by Dahlgren et al. (2001). Therefore, assuming consistent rates of mtDNA evolution across dorvilleids, each of these methane seep clades probably represents separate and distinct species.

In many marine settings, only a single dorvilleid species is present (reviewed in Thornhill et al. 2009). Despite this, instances of multiple co-occurring species have occasionally been previously observed. Smith and Baco (2003) report finding 45 different dorvilleid species on whale falls on the California margin. Wiklund et al. (2009) document the sympatric occurrence of five dorvilleid species – including *Ophryotrocha craigsmithi*, *O. eutrophila*, *O. maculata, O. scutellus*, and *Palpiphitime lobifera* – on an experimental whale fall in the northeast North Atlantic Ocean. Similarly, six dorvilleid species – including *Dinophilus gyrocilatius*, *Ophryotrocha hartmanni*, *O. labronica*, *O. puerilis*, an unidentified *Ophryotrocha* sp., and *Schistomeringos rudolphii* – occurred together in La Spezia Harbor, Italy, with the abundance of each species varying seasonally (Prevedelli et al. 2005). Such cases provide precedents for diverse dorvilleid communities in sulfidic environments. Methane seeps along the NE margin host highly diverse dorvilleid communities, with at least 12 putative sympatric species.

Formal description of the new methane seep taxa is part of a larger project and will be the subject of future reports. However, the lack of morphological variation between certain lineages has stymied traditional taxonomic approaches. Clades, such as *Ophryotrocha* Seep 1 versus Seep 2, *Ophryotrocha* Seep 3 versus 4 versus 5, and *Parougia* Seep CA versus OR were indistinguishable morphologically during shipboard sorting (unpub. data), yet these taxa were well differentiated on both the Cyt *b* and (to a lesser degree) *16S* phylogenies. Such examples of putative cryptic speciation may be common among dorvilleids, including species of *Ophryotrocha*. For instance, although many *Ophryotrocha* lineages examined by Dahlgren et al. (2001) are morphologically similar, breeding experiments attempting to cross hybridize these different lineages have failed to yield viable offspring, suggesting that these taxa were reproductively isolated and were therefore different species according to the biological species concept (Åkesson 1978).

### Establishment and evolution of dorvilleids at methane seeps

Based on the proposed *16S* phylogeny, including methane-seep and nonseep dorvilleids, the ability to inhabit seeps appears to have evolved independently four or more times in this annelid group. Seep *Ophryotrocha* and other taxa fall within the larger phylogeny of nonseep dorvilleids (this paper, Struck et al. 2006; Eibye-Jacobsen and Kristensen 1994). This broader phylogenetic perspective indicates that the ancestor of this clade was likely a nonseep dwelling organism and colonization of seeps occurred multiple times during dorvilleid evolution. For instance, *Ophryotrocha* Seep 1 and Seep 2 are intermingled with various hermaphroditic nonseep *Ophryotrocha*. This phylogenetic position also suggests that the reproductive mode of *Ophryotrocha* Seep 1 and 2 is simultaneous hermaphrodism; however, reproductive mode has not been determined for any of the seep species discussed here. The intermingling of seep and nonseep *Ophryotrocha* indicates that dorvilleids either colonized seep environments multiple times in independent events or have moved in and out of seep environments throughout evolutionary history. Determination of the number of instances where dorvilleid species moved from nonseep habitats into cold seeps requires more exhaustive sampling.

Abundance and diversity of methane-seep dorvilleids suggest that some dorvilleid taxa, such as *Ophryotrocha* and *Parougia*, are preadapted to life at seeps. Notably, some of the closest nonseep relatives to seep-dwelling *Ophryotrocha*, such as *O. adherens* and *O. hartmanni*, are able to survive in marginal, sulfidic, and/or organically enriched environments that are inhospitable to most metazoans (reviewed in Thornhill et al. 2009). Success at marginal and polluted habitats presumably includes mechanisms for detoxifying, tolerating, or avoiding toxic chemicals such as sulfides. Life at seeps presents similar physiological challenges to survival in polluted marine environments, including low levels of dissolved oxygen and high concentrations of hydrogen sulfide (see Introduction). As a result, the finding of intermingled seep and nonseep lineages within the *16S* phylogeny fits within the context of ophryotrochan biology. Adaptation to life in marginal habitats may have preadapted certain *Ophryotrocha* spp. to colonize and succeed at methane seeps, as well as in sulfidic sediments from other environments (e.g., Smith et al. 1998; Mullineaux et al. 2003).

A primary underlying question in the diversification of dorvilleids at seeps is: how do so many confamilial taxa co-exist in this ecosystem? Here, and in previous studies (Levin et al. 2003), it was hypothesized that stressful conditions (e.g., low dissolved oxygen, high-concentrations toxic sulfide) allowed dorvilleids to exploit an environment that was inhospitable to most taxa. Data presented here are consistent with the evolutionary-release hypothesis of Levin et al. (2003). At the hydrocarbon seeps of Hydrate Ridge and Eel River, a high genetic diversity and abundance of dorvilleids were encountered (Levin et al. 2003, 2010; this study). However, no single ecological factor definitively distinguished all species. Preliminary examinations of geographic, habitat, sediment-depth, and dietary differences between taxa suggested that, in many instances, dorvilleid clades were ecologically differentiated from one another through specialization on different resources. Recent isotope and fatty acid analyses of dorvilleds from Eel River and Hydrate Ridge support diet partitioning (Thurber et al. 2012; Levin et al. unpublished). Differences in geographic range (e.g., *Parougia* Seep CA vs. Seep OR), habitat affiliation, depth of sediment, sulfide tolerance (e.g., *P. oregonensis* and *Exallopus* Seep have similar diets, but different sediment distributions), and diet are hypothesized to reduce resource competition between taxa. Such niche partitioning within the environment allows for co-existence of ostensibly similar taxa. High dorvilleid abundance and diversity at whale falls (Smith and Baco 2003) and hydrothermal-vent sediments (Levin et al. 2009) may also relate to release from competition and niche specialization. On the basis of the phylogenetic framework and ecological data presented here, more rigorous investigation of this hypothesis in future studies would be worthwhile.

## Supporting Information

**Table S1.** Haplotype designations, GenBank accession numbers, numbers of samples collected, and collection sites for the dorvilleid annelids examined in this study. The number of haplotypes or determined sequences for haplotypes or dorvilleid clades is given in brackets.
