# Supplementary material for: Adaptive radiation in extremophilic Dorvilleidae (Annelida): diversification of a single colonizer or multiple independent lineages?
Source: Ecol Evol. 2012 Jul 16;2(8):1958–70. doi: 10.1002/ece3.314 (PMC3433998; doi:10.1002/ece3.314)
Supplement: Supplementary file 1 [file ece30002-1958-SD1.doc]

**Supplemental Table 1:** Haplotype designations, GenBank accession numbers, numbers of samples collected and collection sites for the dorvilleid annelids examined in this study. Number of haplotypes or determined sequences for haplotypes or dorvilleid clades are given in brackets.

| *Dorvilleid Taxon/ Clade^1^* | *Concatenated Haplotype Name*  *(43 haplotypes)* | *Cyt b Haplotype Name*  *(41 haplotypes)* | *Cyt b Accession Number* | *16S Haplotype Name*  *(18 haplotypes)* | *16S Accession Number* | *Collection Sites^2^* |
| --- | --- | --- | --- | --- | --- | --- |
| *Ophryotrocha* Seep 1 | 1a (n=1) | 1 (n=1) | XX00000 | a (n=2) | XX00000 | ER-NR |
| (n=6 sequences) | 2b (n=3) | 2 (n=3) |  | b (n=3) |  | ER-SR |
|  | 3a (n=1) | 3 (n=1) |  | a |  | ER-SR |
|  | 4c (n=1) | 4 (n=1) |  | c (n=1) |  | ER-SR |
| *Ophryotrocha* Seep 2 | 5d (n=1) | 5 (n=2) |  | d (n=10) |  | ER-NR |
| (n=11 sequences) | 5e (n=1) | 5 |  | e (n=1) |  | ER-SR |
|  | 6d (n=5) | 6 (n=5) |  | d |  | ER-NR,SR |
|  | 7d (n=1) | 7 (n=1) |  | d |  | ER-NR |
|  | 8d (n=1) | 8 (n=1) |  | d |  | ER-NR |
|  | 9d (n=1) | 9 (n=1) |  | d |  | ER-NR |
|  | 10d (n=1) | 10 (n=1) |  | d |  | ER-NR |
| *Ophryotrocha* Seep 3 | 11f (n=1) | 11 (n=1) |  | f (n=2) |  | HR-E |
| (n=2 sequences) | 12f (n=1) | 12 (n=1) |  | f |  | HR-E |
| *Ophryotrocha* Seep 4 | 13g (n=2) | 13 (n=2) |  | g (n=6) |  | ER-NR |
| (n=12 sequences) | 14h (n=3) | 14 (n=3) |  | h (n=3) |  | ER-SR |
|  | 15i (n=3) | 15 (n=3) |  | i (n=3) |  | ER-SR |
|  | 16g (n=4) | 16 (n=4) |  | g |  | ER-SR |
| *Parougia oregonensis* Clade 1 | 17j (n=5) | 17 (n=5) |  | j (n=13) |  | ER-NR; HR-N,S,E |
| (n=13 sequences) | 18j (n=1) | 18 (n=1) |  | j |  | HR-N |
|  | 19j (n=5) | 19 (n=5) |  | j |  | HR-N,S |
|  | 20j (n=1) | 20 (n=1) |  | j |  | HR-E |
|  | 21j (n=1) | 21 (n=1) |  | j |  | HR-S |
| *Parougia oregonensis* Clade 2 | 22k (n=1) | 22 (n=3) |  | k (n=2) |  | HR-S |
| (n=4 sequences) | 22l (n=2) | 22 |  | l (n=2) |  | ER-SR |
|  | 23k (n=1) | 23 (n=1) |  | k |  | HR-N |
| *Parougia* Seep Clade CA | 24m (n=9) | 24 (n=9) |  | m (n=15) |  | ER-NR,SR;HR-S |
| (n=15 sequences) | 25m (n=4) | 25 (n=4) |  | m |  | ER-NR |
|  | 26m (n=1) | 26 (n=1) |  | m |  | ER-NR |
|  | 27m (n=1) | 27 (n=1) |  | m |  | ER-SR |
| *Parougia* Seep Clade OR | 28n (n=23) | 28 (n=24) |  | n (n=38) |  | HR-N,S,E |
| (n=39 sequences) | 28o (n=1) | 28 |  | o (n=1) |  | HR-S |
|  | 29n (n=2) | 29 (n=2) |  | n |  | HR-S |
|  | 30n (n=2) | 30 (n=2) |  | n |  | HR-S |
|  | 31n (n=3) | 31 (n=3) |  | n |  | HR-S |
|  | 32n (n=2) | 32 (n=2) |  | n |  | HR-S |
|  | 33n (n=3) | 33 (n=3) |  | n |  | HR-E |
|  | 34n (n=1) | 34 (n=1) |  | n |  | HR-S |
|  | 35n (n=1) | 35 (n=1) |  | n |  | HR-S |
|  | 36n (n=1) | 36 (n=1) |  | n |  | HR-S |
| *Ophryotrocha* Seep 5 | 37p (n=3) | 37 (n=3) |  | p (n=5) |  | ER-NR |
| (n=5 sequences) | 38p (n=2) | 38 (n=2) |  | p |  | ER-SR |
| *Exallopus* Seep | 39q (n=3) | 39 (n=3) |  | q (n=20) |  | ER- SR; HR-S |
| (n=20 sequences) | 40q (n=17) | 40 (n=17) |  | q |  | ER-NR,SR; HR-N,S |
| *Pinniphitime* Seep |  | 41 (n=3) |  | -^3^ |  | ER-NR; HR-N,S |
| *Pseudophryotrocha* Seep |  | -^4^ |  | r (n=1) |  | ER-NR |

^1^: Corresponding to the phylogenies presented in Figs. 2–4

^2^: Collection site abbreviations are as follows: ER = Eel River, California, NR = North Ridge, SR = South Ridge; HR = Hydrate Ridge, Oregon, N = North, S = South, E = East

^3^: Sample failed to sequence for 16S

^4^: Sample failed to sequence for Cyt b
